# The Transcription Factor SOX18 Inhibitor Small Molecule 4 Is a Potential Treatment of Cancer‐Induced Lymphatic Metastasis and Lymphangiosarcoma

**DOI:** 10.1002/cnr2.70110

**Published:** 2025-01-10

**Authors:** Katja K. Koll, Martin M. Zimmermann, Patrick A. Will, Ulrich Kneser, Christoph Hirche

**Affiliations:** ^1^ Department of Hand, Plastic and Reconstructive Surgery, Microsurgery Burn Centre BG Klinik Ludwigshafen Ludwigshafen Germany; ^2^ Medical Faculty of the University Heidelberg Heidelberg Germany; ^3^ Department of Plastic and Hand Surgery Faculty of Medicine and University Hospital Carl Gustav Carus, TU Dresden Dresden Germany; ^4^ Department of Plastic, Hand, and Reconstructive Microsurgery, BG Unfallklinik Frankfurt Am Main Affiliated Hospital of Goethe‐University Frankfurt am Main Germany

**Keywords:** cancer‐induced lymphatic metastasis, lymphangiosarcoma, lymphatic endothelial cells, MO‐LAS, Sm4, SOX18 inhibitor

## Abstract

**Background:**

Malignant tumors release growth factors, promoting lymphangiogenesis in primary tumors and draining sentinel lymph nodes, ultimately facilitating lymph node metastasis. As a malignant lymphatic tumor entity, lymphangiosarcomas are characterized by low survival rates and limited treatment options. The transcription factor SOX18 plays a crucial role in both lymphatic endothelial cell differentiation and cancer‐induced lymphangiogenesis.

**Aims:**

In this in vitro study, we investigated the potential therapeutic effect of a small molecule called Sm4, which inhibits SOX18, on lymphatic endothelial and lymphangiosarcoma cells in vitro.

**Methods and Results:**

Human dermal lymphatic endothelial cells (HDLECs), lymphangiosarcoma cells (MO‐LAS), and other endothelial cell lines were cultured. We found that *Sox18* exhibited high mRNA expression levels in both HDLEC and MO‐LAS. Sm4 treatment decreased the *Sox18* expression level at the mRNA and protein levels in both HDLEC and MO‐LAS significantly, a phenomenon confirmed through immunofluorescence images. Additionally, Sm4 treatment suppressed the expression of key lymphatic phenotype markers (*Prox1*, *Flt4*, and *Lyve1*) and hindered migration in both HDLEC and MO‐LAS, all while maintaining cell viability.

**Conclusion:**

These findings suggest that targeting SOX18 with Sm4 may hold potential as a therapeutic strategy for lymphangiosarcoma and cancer‐induced lymphatic metastasis. Further in vitro studies are warranted to investigate the mechanisms and conduct dose–response analyses to evaluate Sm4's potential as a targeted therapy for lymphangiosarcoma and cancer‐induced lymphangiogenesis in the future.

## 1 Introduction

The leading cause of cancer‐related deaths is cancer metastasis. While numerous studies have explored the mechanisms of tumor metastasis through the bloodstream to distant organs, a substantial portion of epithelial cancers initially undergo metastatic growth by spreading through lymphatic vessels to their draining lymph nodes [1]. Malignant tumors release growth factors like vascular endothelial growth factor C (VEGF‐C), promoting the expansion of lymphatic vessels (lymphangiogenesis) in primary tumors and draining sentinel lymph nodes, facilitating lymph node metastasis [2, 3, 4]. Recent evidence suggests that lymphatic vessels not only act as passive channels for tumor spread but may actively enhance tumor cell recruitment to lymph nodes: Tumor‐draining lymphatics could facilitate tumor spread through increased pumping and lymph flow, often influenced by VEGF‐C [5, 6, 7]. Additionally, the lymphatic endothelium serves as a niche for cancer cells, providing a specialized microenvironment that supports tumor cell survival and potentially their proliferation. Metastatic tumors developing in lymphatic vessels between the primary tumor and the draining lymph node suggest that the lymphatic endothelium could offer a protective microenvironment, shielding cancer cells from immune attacks, supplying necessary nutrients, and facilitating their migration to distant sites [8]. Due to its potential and its role in cancer spread, the lymphatic endothelium has emerged as a compelling target for innovative cancer therapies.

Furthermore, the endothelial cells themselves have the potential to undergo mutations leading to cancer. Angiosarcomas, rare malignant vascular tumors originating from endothelial cells, pose significant challenges due to their low 5‐year survival rate of approximately 41%–43% and the lack of effective therapeutic options [9]. Angiosarcomas can develop in various parts of the body, but they are frequently observed in the head, neck, skin, and breast regions [10]. Angiosarcomas with lymphatic differentiation are termed lymphangiosarcomas. They exhibit increased expression of lymphatic phenotype key markers such as VEGFR3, PROX1, LYVE1, and podoplanin [11, 12, 13]. Given the high lethality associated with these tumors and the lack of understanding of their pathophysiology, it appears crucial to explore new treatment avenues.

Transcription factor SRY‐related HMG‐box 18 (SOX18) plays a critical role as an essential regulator of endothelial cell differentiation [14, 15] and endothelial cell barrier [16] in the development of cardiovascular and lymphatic vessels. Notably, SOX18 directly induces the expression of *Prox1*, *Flt4*, and *podoplanin*, promoting the differentiation of lymphatic endothelial cells [15]. SOX18 is associated with advanced tumor progression and considerably influences tumor cell regulation [17].

While SOX18 has been broadly studied in various cancers such as skin [18, 19], stomach [20], liver [21], breast [22], lung [23, 24], cervical, and ovarian cancer [25, 26], its role in lymphangiosarcoma remains unexplored. Furthermore, SOX18 has been implicated in cancer‐related lymphangiogenesis, and its suppression has shown promise in inhibiting tumor metastasis [27]. However, the extensive need to discover new factors, molecular mechanisms, and promising targets for antilymphangiogenic treatment still remains [28].

Recent studies have highlighted the potential of developing specific inhibitors for transcription factors. A notable advancement in this field is the work of Fontaine et al. [29], who reported the development of a specific inhibitor, small molecule 4 (Sm4), targeting the SOX18 transcription factor.

Sm4, an analog of salicylic acid, is a synthetic inhibitor designed to interfere with the SOX18 HMG DNA‐binding domain and disrupt HMG‐dependent protein–protein interactions (PPI) with RBPJ. Mapping the putative binding site of Sm4 onto the RBPJ/SOX18 complex positions the inhibitor directly in the SOX18 DNA‐binding region of helix 3 and the C‐terminal tail, opposite its main protein–protein interface. This suggests that Sm4 binding could perturb both protein–protein and protein–DNA interactions. In contrast, no interference was observed with the SOX18‐MEF2C interaction, indicating that Sm4 exhibits some level of specificity as a PPI disruptor. While the inhibition of SOX18‐DNA‐binding activity lacks specificity, selective inhibition of specific SOX18‐dependent PPIs could lead to a targeted transcriptional blockade of a subset of SOX18 direct target genes. The development and optimization of small molecules with selectivity for specific PPIs may provide an alternative strategy for targeting transcription factor activity [29].

Studies conducted in zebrafish larvae have demonstrated that Sm4 suppressed genes downstream of *Sox18*, thereby interfering with vascular development [30]. Notably, in a mouse model of breast cancer, treatment with Sm4 has shown a reduction in tumor vascular density and metastatic spread through the lymphatic system. Specifically, the Sm4 treatment led to a significant 65% reduction in the density of tumor‐associated lymphatic vessels, coupled with a remarkable 70% decrease in the number of lymphatic endothelial cells [30].

By elucidating the molecular mechanisms underlying the pathogenesis of lymphangiosarcoma and cancer‐induced lymphatic metastasis, it becomes evident that targeted therapeutic drugs, such as Sm4, hold increasing importance. Here, we investigated the potential therapeutic effect of inhibiting SOX18 via Sm4 pathway in lymphatic endothelial cells and lymphangiosarcoma cells in vitro to evaluate its role as a treatment target.

## 2 Materials and Methods

### 2.1 Cell Culture

Human dermal lymphatic endothelial cells (HDLECs) derived from juvenile foreskin, human umbilical vein endothelial cells (HUVEC), and human dermal blood endothelial cells (HDBEC) were purchased from PromoCell GmbH (Heidelberg, Germany). These cells were cultured in a specific endothelial cell growth medium MV (C‐22020, PromoCell). For HDLEC culturing, corresponding endothelial cell growth medium MV2 supplemental mix (C‐39226, PromoCell), and for HUVEC culturing, supplemental mix C‐39215 (PromoCell) plus 2 mM glutamine were added.

The lymphangiosarcoma cell line MO‐LAS was obtained from Shigeo Nishiyama, Kyoto University [31]. Cells were cultured in RotiCell DMEM high glucose (Carl Roth; Karlsruhe, Germany) added with 10% fetal bovine serum advanced (FBS‐11A, Capricorn Scientific GmbH; Ebsdorfergrund, Germany) and 1% penicillin–streptomycin (Thermo Fisher Scientific, Waltham, USA).

Each cell culture medium was changed three times a week. All cells were cultured in an incubator at 37°C with 5% CO2. Cells were split at 80% confluency and were passaged at a 1:3 ratio with TrypLE Express, phenol red (12 605 028, Thermo Fisher Scientific, Waltham, USA). For all experiments, cells in passages 5–7 with a density of 15.000 cells/cm^2^ were used.

### 2.2 Sm4 Application

Sm4, SML1999‐5 mg (Sigma‐Aldrich, Merck; Darmstadt, Germany) was dissolved in 2.5 mL DMSO (Carl Roth; Karlsruhe, Germany). To ensure proper controls, cells received the exact volume of DMSO vehicle corresponding to the amount used to solubilize Sm4, ensuring that the final concentration of DMSO in all treatments, including controls, was consistent. The percentage of DMSO used depended on the concentration (ranging from 0.0076% to 4.56%) and was consistent for both treated and control groups. To determine the concentration response of Sm4 in HDLEC, we referred to a study by Fontaine et al. [29] where the half‐maximal inhibitory concentration (IC50) of cell‐based luciferase SOX18‐dependent transactivation in fibroblasts was defined as 5.2 μM. We initiated our experiments at this concentration of 5.2 μM and gradually increased the concentration in multiples (5‐fold to 26 μM, 10‐fold to 52 μM, 15‐fold to 78 μM, 20‐fold to 104 μM, and so forth) until a significant reduction in *Sox18* gene expression was observed in the HDLEC or MO‐LAS. The test series was applied to the cells for a duration of 24 h. To explore potential effects over a longer period, we extended the application time to 48 h to assess whether there was a greater impact on the *Sox18* mRNA expression level.

### 2.3 qPCR

RNA was isolated using the NucleoSpin RNA kit (Macherey‐Nagel; Düren, Germany) according to the manufacturers' protocols. cDNA synthesis from up to 0.5 μg RNA was performed with Maxima H Minus First Strand cDNA Synthesis Kit (Thermo Fisher Scientific). SYBR Green (Thermo Fisher Scientific) on a LightCycler 480 (Roche Diagnostics, Basel, Switzerland) was used for quantitative real‐time PCR. Gapdh mRNA expression levels were measured to normalize the gene expression using the comparative CT (ΔΔCT) method. At least three replicates per group were analyzed. qPCR primers are listed in Table S1.

### 2.4 Immunoblot Analysis

For immunoblot analysis, protein preparations in RIPA lysis buffer with protease inhibitor (Thermo Fisher Scientific) were mixed with 5 × Laemmli loading buffer and heated to 95°C for 10 min. Equal amounts of protein were subjected to electrophoresis and transferred to polyvinylidene fluoride (PVDF) membranes (Rotiphorese PROclamp mini, Carl Roth; Karlsruhe, Germany). The primary applied antibody was anti‐SOX18 (monoclonal, 1:400, Santa Cruz, sc‐166 025). The mouse monoclonal anti‐GAPDH (monoclonal, 1:3000, Fitzgerald, 10R‐G109a) was used as a loading control. Western blot bands were visualized by using Azure 400 (Azure Biosystems, California, USA) and quantified using ImageJ.

### 2.5 Immunofluorescence Staining

Immunofluorescence staining was performed with 2 × 10^4^ seeded cells per cm^2^ in 24‐well plates. Cells were fixed in 4% PFA for 20 min, permeabilized in 0.3% Triton X‐100 for 20 min, and blocked with 3% BSA for at least 1 h at room temperature. Each step was followed by repeated washing in PBS. Slides were then serially incubated with primary and secondary antibodies. Negative controls were included where the primary antibody was omitted. The primary antibody anti‐SOX18 (1:200, Santa Cruz, sc‐166 025) was used. Alexa Flour 488 donkey antimouse (Dianova, 715–545‐150) was the secondary antibody with a 1:400 dilution. Slides were covered with a mounting solution containing DAPI (Vectashield H‐1200, Vector, Newark, USA) to stain nuclei. Cells were visualized by using an AXIO Imager. M2 microscope (Zeiss; Oberkochen, Germany). For each sample, three randomly chosen sites of the capsule were digitalized at a 20 × magnification.

### 2.6 Cell Viability Assay

To analyze cell viability following incubation with Sm4, we employed an MTT viability assay. MO‐LAS and HDLEC were exposed to different levels of Sm4 concentration for 24 h. Afterwards, 10 μL of MTT (3‐(4,5‐Dimethylthiazol‐2‐yl)‐2,5‐diphenyl‐tetrazolium bromide) (Serva; Heidelberg, Germany) was added for 3 h. Following the addition of 100 μL of acidified isopropanol per well, the color intensity was measured using an ELISA plate reader (Glomax Discover; Promega, Germany) at a wavelength of 560 nm for analysis and 600 nm for reference. The proliferation rate was determined using GraphPad Prism 8 software, and the results were expressed as a percentage of the color intensity relative to the control group.

### 2.7 Cell Proliferation Assay

For assessment of proliferation, HDLEC and MO‐LAS were plated on a 96‐well plate and treated with Sm4 or DMSO, respectively. A colorimetric BrdU incorporation immunoassay (BrdU Cell Proliferation Kit 2750, Merck; Darmstadt, Germany) was used following the manufacturer's instructions. The absorbance was measured at 450 nm with a plate reader (Glomax Discover; Promega, Germany).

### 2.8 Scratch Assay

To analyze the migration capacity, 3 × 10^4^ HDLEC or MOLAS were plated per cm^2^ on 6‐well plates. When the cells reached 90%–100% confluency, the complete medium was removed, and the cells were subjected to a 7‐h starvation period in serum‐free medium. Following starvation, the cell monolayer was scraped in a straight line using a 200 μL pipette tip to create a scratch. Subsequently, cells were treated with Sm4, DMSO only or were left unstimulated. To analyze the migration, the same scratch area was captured with a Zeiss Axio Observer Z.1 (Zeiss; Germany) microscope after 0 and 24 h. The wound area was calculated as cell‐free area at 0, 24 h/cell‐free area at 0 h, and the cell‐free area was quantified using ImageJ, as described in previous studies [32].

### 2.9 ELISA

Protein samples were isolated using RIPA lysis buffer containing a Protease Inhibitor (Thermo Fisher Scientific), and their concentrations were determined using the BCA assay (Pierce BCA Protein Assay Kit, Thermo Fisher Scientific). The LYVE1 ELISA kit (ELH‐LYVE1‐1 from RayBiotech) was utilized following the manufacturer's instructions. Appropriate sample dilutions were determined through preliminary tests. All samples were analyzed in duplicates, with corresponding negative controls. Standard curves were generated, and absorbance at 450 nm was measured to quantify and compare protein levels across different conditions.

### 2.10 Statistical Analysis

All data are presented as mean ± SEM. Acquired data was statistically analyzed by means of Graph Pad Prism 8 (GraphPad Software Inc., USA). An unpaired, two‐tailed Student's *t*‐test was used to determine statistical significance between two groups, and ANOVA was utilized for comparisons across multiple groups. *P* values less than 0.05 were considered statistically significant.

## 3 Results

### 3.1 Both Human Dermal Lymphatic Endothelial Cells and Lymphangiosarcoma Cells (MO‐LAS) Demonstrated Elevated Levels of Sox18 mRNA Expression

**FIGURE 1:**
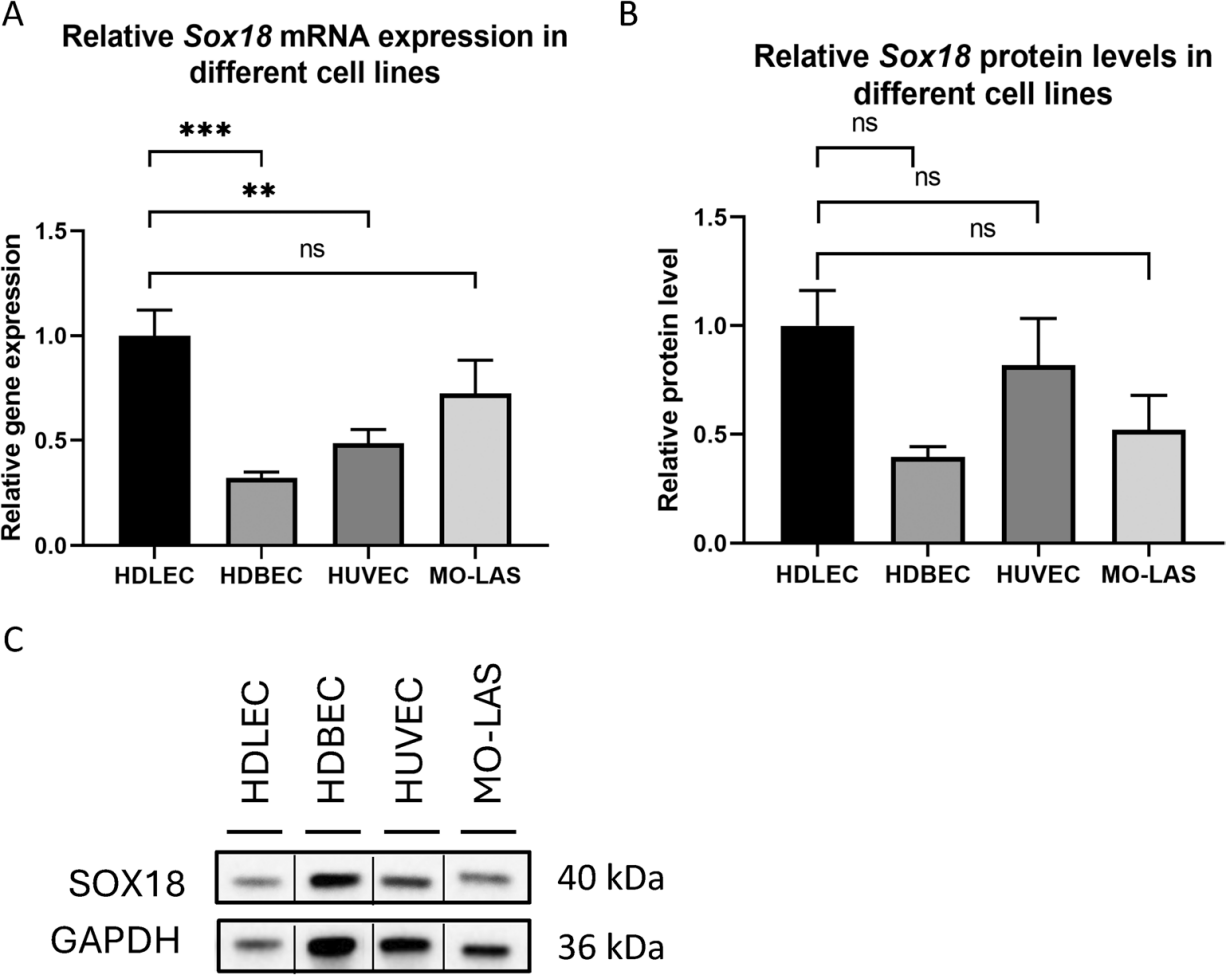
*Sox18* expression and protein level in HDLEC and the MO‐LAS. (A) Relative mRNA expression of *Sox18* in human dermal lymphatic endothelial cells (HDLEC) was significantly higher related to human dermal blood endothelial cells (HDBEC) and human umbilical vein endothelial cells (HUVEC), and equally high as in lymphangiosarcoma cells (MO‐LAS) examined by qPCR. GAPDH was used as a control gene. (B, C) Western blot analysis with densitometric quantification illustrating the level of SOX18 protein (40 kDa) in HDLEC compared with HDBEC, HUVEC, and MO‐LAS. The vertical lines in the western blot denote that the blot has been spliced to remove the duplicate lanes. GAPDH was used as a loading control (36 kDa). Western blot analysis shown is representative of independent experiments. Data are shown as mean ± SEM. All experiments were performed using technical and biological replicates. ns = *p* > 0.05—not significant, ** = *p* < 0.01, *** = *p* < 0.001.

To assess the relative *Sox18* expression across different cell lines, we compared human dermal lymphatic endothelial cell (HDLEC) with other cell types, including HUVEC, HDBEC, and the MO‐LAS cell line. The mRNA expression of *Sox18* in HDLEC was 68% higher than in HDBEC (1.00 ± 0.07 vs. 0.32 ± 0.02, *p* = 0.0002, *n* = 6) and 51% higher in HUVEC (1.00 ± 0.07 vs. 0.49 ± 0.04, *p* = 0.0016), whereas no statistically significant difference was observed between MO‐LAS and HDLEC (1.00 ± 0.07 vs. 0.72 ± 0.09, *p* = 0.054) (Figure 1A). Immunoblot analysis also revealed that HDLEC exhibited the highest level of SOX18 protein among the tested cell lines (HDLEC 1.00 ± 0.12 vs. HDBEC = 0.4 ± 0.03, *p* = 0.0613; HDLEC 1.00 ± 0.12 vs. HUVEC 0.82 ± 0.15, *p* = 0.69; HDLEC 1.00 ± 0.12 vs. MOLAS 0.52 ± 0.11, *p* = 0.12), although the differences were not statistically significant (Figure 1B,C).

### 3.2 Dose Dependency of Sm4 in HDLEC

**FIGURE 2:**
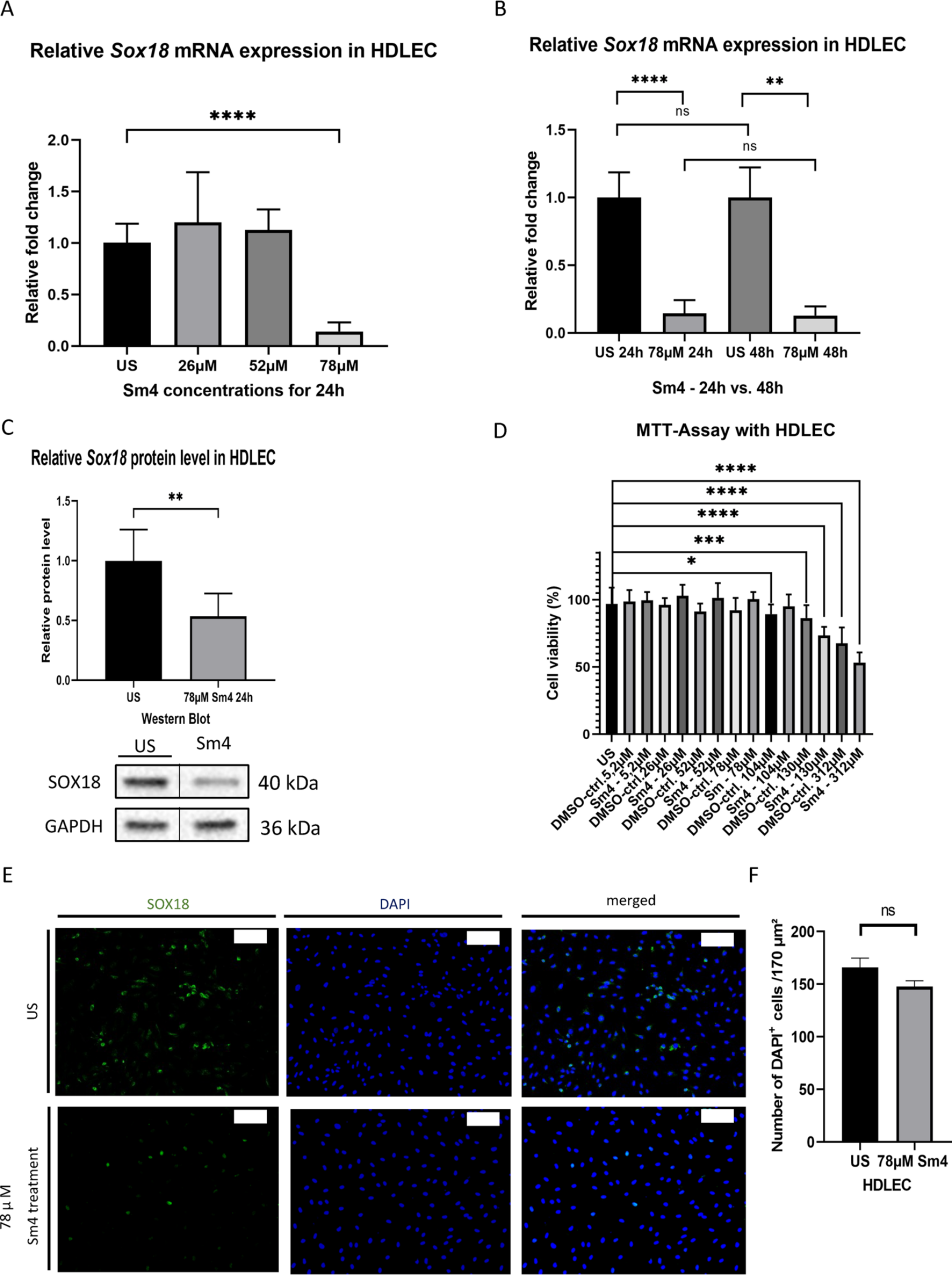
Dose dependency of Sm4 in HDLEC. (A) Following a 24‐h treatment of HDLEC with various levels of Sm4 concentration, there was an alteration in the relative mRNA expression of Sox18, with a significant difference observed at a concentration of 78 μM, as indicated by qPCR analysis. (B) HDLEC subjected to a 78 μM Sm4 treatment for 24 h displayed a comparable effect on the relative *Sox18* mRNA expression to that observed after a 48‐h treatment, as determined by qPCR analysis. Medium was renewed after 24 h. (C) Efficiency of SOX18 reduction was demonstrated on the protein level (40 kDA) by Western blot and densitometric quantification in HDLEC after treatment with 78 μM Sm4 for 24 h. The vertical lines in the western blot denote that the blot has been spliced to remove the triplicate lanes. GAPDH was used as a loading control (36 kDa). Western blot analysis shown is representative of an independent experiment. (D) The viability of human dermal lymphatic endothelial cells (HDLEC) remained unchanged even at a higher concentration of Sm4 for a 24‐h period, up until a concentration of 130 μM, as determined through the MTT assay. (E) Representative immunofluorescence staining images showing SOX18 (green) and DAPI (blue) in HDLEC after a treatment of 78 μM Sm4 for 24 h. Scale bars: 100 μm. (F) The number of DAPI‐positive HDLEC cells per 170 μm^2^ did not change after 78 μM Sm4 treatment compared to the unstimulated cells. Data are shown as mean ± SEM. All experiments were performed using technical and biological replicates. ns = *p* > 0.05—not significant, * = *p* < 0.05, ** = *p* < 0.01, *** = *p* < 0.001, **** = *p* < 0.0001.

Starting from a concentration of 5.2 μM and progressively increasing the concentration up to a 15‐fold of 5.2 μM (78 μM), we observed a noteworthy 86% reduction of the relative *Sox18* mRNA level in HDLEC after a 24‐h exposure (unstimulated (US) 1.00 ± 0.05 vs. 78 μM Sm4 0.14 ± 0.03, *p* < 0.0001) (Figure 2A). Prolonging the exposure time to 48 h did not result in any further reduction in relative *Sox18* mRNA expression (24‐h treatment 0.14 ± 0.03 vs. 48‐h treatment 0.13 ± 0.04, *p* = 0.9987) (Figure 2B). Immunoblotting analysis demonstrated that treatment with 78 μM of Sm4 for 24 h led to a 46% reduction in relative SOX18 protein levels compared to unstimulated cells (US 1.00 ± 0.11 vs. 78 μM of Sm4 0.54 ± 0.07, *p* = 0.0034) (Figure 2C). Upon investigating cell viability, a significant decline was noted with a concentration increase of 130 μM (equivalent to sixtyfold of 5.2 μM), demonstrating a substantial reduction (mean difference 23.52% ± 2.97%, *p* < 0.0001). However, viability did not show significant changes with the concentration of 78 μM (mean difference 3.52% ± 2.68%, *p* = 0.853) (Figure 2D). Reduced SOX18 protein abundance in Sm4 treated with 78 μM HDLEC was shown by immunofluorescence staining (Figure 2E). Cell counting revealed no difference in the number of HDLEC between the 78‐μM Sm4 treatment group and the control group (mean difference 18.33 ± 10.4, *p* = 0.1526).

### 3.3 Sm4 Suppressed the Expression of Lymphatic Phenotype Key Markers in HDLEC and Reduced Cell Migration and Proliferation

**FIGURE 3:**
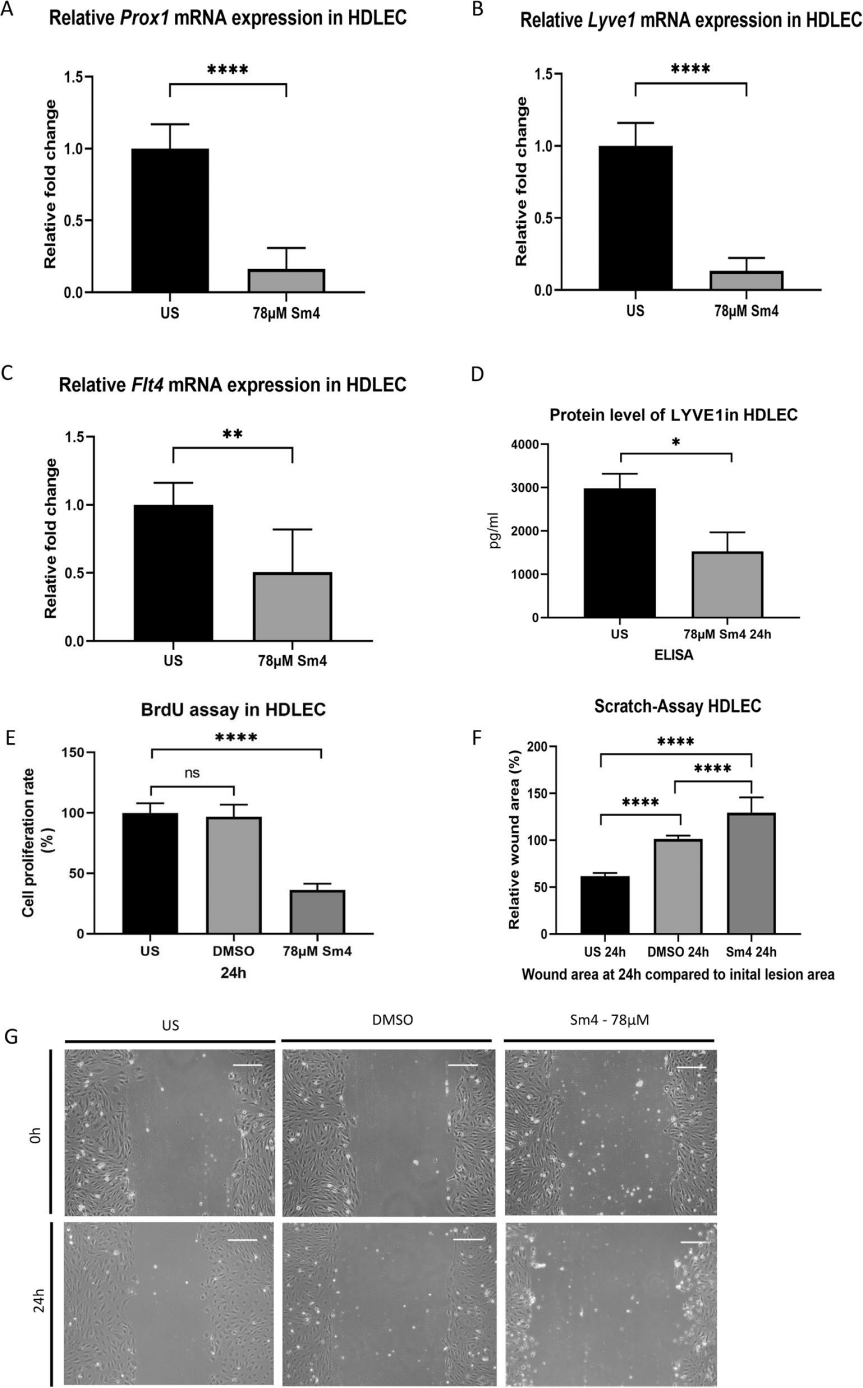
Suppressed lymphatic phenotype key markers and reduced cell migration and proliferation of HDLEC after Sm4 treatment. (A–C) Relative mRNA expression of *Prox1* (A), *Lyve1* (B), and *Flt4* (C) in human dermal lymphatic endothelial cells (HDLEC) was diminished after a 78‐μM Sm4 treatment for 24 h were shown by qPCR. (D) The LYVE1 protein level was reduced after treatment with 78 μM Sm4 for 24 h, as tested by ELISA. (E) Reduced proliferation of HDLEC with Sox18 deletion after 78 μM Sm4 for 24 h compared with controls was measured by BrdU incorporation proliferation assay. (F, G) Migration of HDLEC treated with 78 μM Sm4 or under baseline conditions was quantified as wound area after 24 h. Representative pictures are shown directly after the scratch and 24 h later. Scale bars: 100 μm. Data are shown as mean ± SEM. All experiments were performed using technical and biological replicates. ns = *p* > 0.05—not significant, * = *p* < 0.05, ** = *p* < 0.01, **** = *p* < 0.0001.

We investigated Sm4's impact on key lymphatic phenotype markers in HDLEC. 78 μM Sm4 treatment reduced mRNA levels significantly, including an 84% decrease in *Prospero Homeobox 1* (*Prox1*) expression (1.00 ± 0.07 vs. 0.16 ± 0.06, *p* < 0.0001, *n* = 12), an 87% reduction in *lymphatic vessel endothelial hyaluronan receptor 1* (*Lyve1*) expression (1.00 ± 0.07 vs. 0.13 ± 0.04, *p* < 0.0001, *n* = 12), and a 49% decrease in *Fms‐related tyrosine kinase 4* (*Flt4*) expression (1.00 ± 0.07 vs. 0.51 ± 0.13, *p* = 0.0065) after 24 h (Figure 3A–C). Furthermore, the LYVE1 protein level in HDLEC was significantly decreased following treatment with 78 μM Sm4 (difference between means 1459 ± 374.6, *p* = 0.03).

Cell proliferation, evaluated using a BrdU incorporation immunoassay, revealed a significantly lower proliferation rate in Sm4‐treated HDLEC compared to untreated HDLEC (100.00% ±7.6% vs. 36.3% ± 4.98%, *p* < 0.0001) (Figure 3E). Additionally, cell migration assays demonstrated a significant reduction in migration levels in Sm4‐treated HDLEC compared to the untreated control group, as quantified by the relative wound area after 24 h (difference between the means of US vs. 78 μM Sm4 67.93% ± 5.197%, *p* < 0.0001, the difference between the means of DMSO control vs. 78 μM Sm4 28.34% ± 5.197%, *p* < 0.0001). Additionally, a significant difference was observed between the DMSO treatment and the unstimulated control (difference between the means of US vs. DMSO control for 78 μM 39.59% ± 5.197%, *p* < 0.0001) (Figure 3F,G). These findings suggest that Sm4 treatment leads to decreased cellular activity in HDLEC.

### 3.4 Dose Dependency of Sm4 in MO‐LAS

**FIGURE 4:**
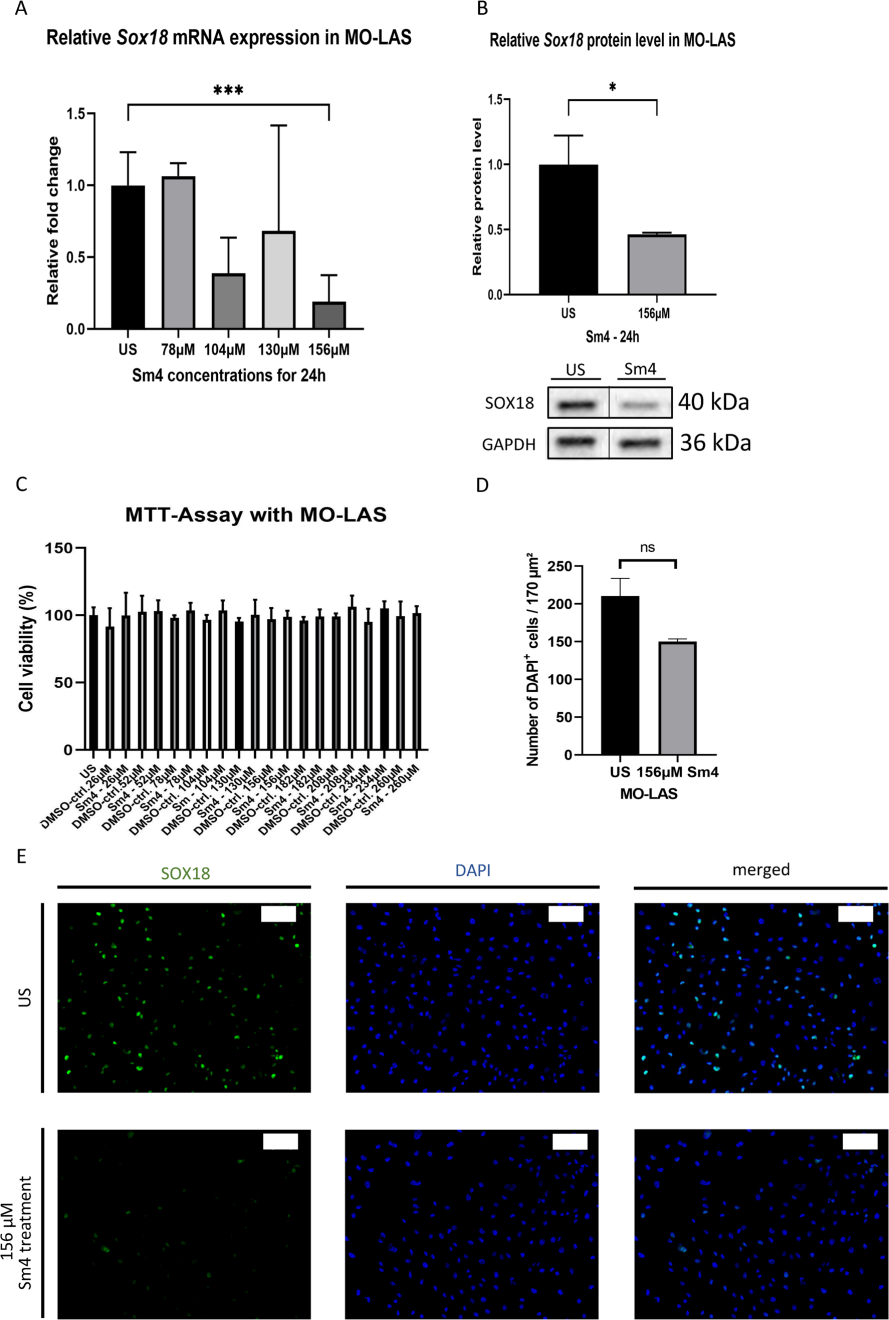
Dose‐dependency of Sm4 in MO‐LAS: (A) Following a 24‐h Sm4 treatment at various levels of Sm4 concentration, the relative mRNA expression of *Sox18* in HDLEC exhibited a notable reduction, significantly at a concentration of 156 μM, as determined through qPCR analysis. GAPDH was used as a control gene. (B) The effectiveness of SOX18 reduction was confirmed at the protein level (40 kDa) through Western blot analysis and densitometric quantification in MO‐LAS cells following a 24‐h treatment with 156 μM Sm4. The vertical lines in the western blot denote that the blot has been spliced to remove the replicate lanes (*n* = 4 for unstimulated MO‐LAS and *n* = 6 for Sm4 stimulated MO‐LAS). GAPDH was used as a loading control. GAPDH was used as a loading control (36 kDa). Western blot analysis shown is representative of independent experiments. (C) The viability of MO‐LAS cells remained unaffected at a higher concentration of Sm4 for 24 h, as demonstrated by the MTT assay. (D) The number of DAPI‐positive MO‐LAS cells per 170 μm^2^ remained unchanged after treatment with 156 μM Sm4 compared to the unstimulated cells. (E) Representative immunofluorescence staining images showing SOX18 (green) and DAPI (blue) in MOLAS after treatment of 156 μM Sm4 for 24 h. Scale bars: 100 μm. Data are shown as mean ± SEM. All experiments were performed using technical and biological replicates. ns = *p* > 0.05—not significant, * = *p* < 0.05, *** = *p* < 0.001.

To determine the effective concentration of Sm4 in MO‐LAS, we assessed the relative *Sox18* mRNA expression levels in MO‐LAS cells. The effective concentration of Sm4 in HDLEC did not show significant alterations in relative *Sox18* mRNA expression levels in MO‐LAS (1.00 ± 0.08 vs. 1.07 ± 0.05, *p* = 0.9963). Increasing the concentration of Sm4 revealed a significant decrease in *Sox18* expression levels in MO‐LAS using a concentration of 156 μM (a thirtyfold of 5.2 μM) (1.00 ± 0.08 vs. 0.19 ± 0.11, *p* = 0.0088, *n* = 12) (Figure 4A). Immunoblotting analysis concordantly demonstrated that treatment with 156 μM of Sm4 for 24 h resulted in a 54% reduction of SOX18 protein levels in MO‐LAS (1.00 ± 0.13 vs. 0.46 ± 0.01, *p* = 0.0482, *n* = 5) (Figure 4B). Cell viability remained unaltered after 24 h with a concentration of 156 μM (mean difference 1.226 ± 2.27, *p* = 0.993), and no significant differences were seen at other concentrations either (Figure 4C). The reduced SOX18 protein abundance was confirmed in Sm4‐treated MO‐LAS, with no significant differences in MO‐LAS cell count (mean difference 60.33 ± 23.77, *p* = 0.0641) (Figure 4D), as shown by immunofluorescence staining after treatment with 156 μM Sm4 (Figure 4E).

### 3.5 Sm4 Suppressed the Expression of Lymphatic Phenotype Key Markers in MO‐LAS and Reduced Cell Migration

**FIGURE 5:**
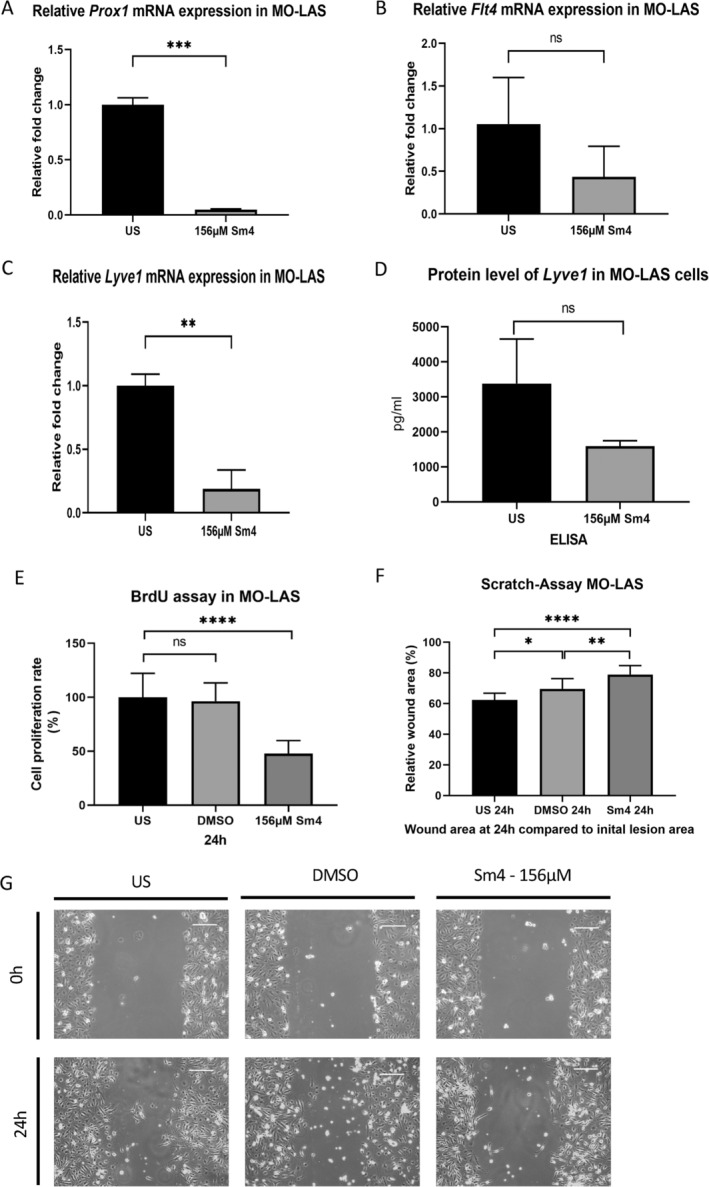
Suppressed lymphatic phenotype key markers and reduced cell migration and proliferation of MO‐LAS after Sm4 treatment. (A–C) After a 24‐h treatment with 156 μM Sm4, qPCR analysis demonstrated a decrease in the relative mRNA expression of *Prox1* (A), *Lyve1* (B), and *Flt4* (C) in MO‐LAS. (D) The LYVE1 protein levels in MO‐LAS cells after treatment with 156 μM Sm4, compared to unstimulated controls, were assessed using ELISA. (E) Reduced proliferation of MO‐LAS cells with SOX18 deletion after 24 h of treatment with 156 μM Sm4, compared to controls, was measured using the BrdU incorporation proliferation assay. (F, G) Reduced migration of MO‐LAS treated with 156 μM Sm4 or under baseline conditions was quantified as wound area after 24 h. Representative pictures are shown directly after the scratch and 24 h later. Scale bars: 100 μm. Data are shown as mean ± SEM. All experiments were performed using technical and biological replicates. ns = *p* > 0.05—not significant, * = *p* < 0.05, ** = *p* < 0.01, *** = *p* < 0.001, **** = *p* < 0.0001.

Sm4 treatment in MO‐LAS cells led to a mostly significant decrease in mRNA levels of *Prox1* (1.00 ± 0.04 vs. 0.05 ± 0.01, *p* = 0.0003, *n* = 5), *Flt4* (1.00 ± 0.22 vs. 0.43 ± 0.16, *p* = 0.0583, *n* = 11), and *Lyve1* (1.00 ± 0.05 vs. 0.19 ± 0.09, *p* = 0.0013, *n* = 6) after 24 h compared to untreated MO‐LAS cells (Figure 5A–C). The LYVE1 protein level in treated MO‐LAS cells was lower compared to controls, but the difference was not statistically significant (1794 ± 0.152, *p* = 0.152) (Figure 5D).

BrdU incorporation immunoassay showed a significantly lowered proliferation rate in Sm4‐treated MO‐LAS compared to untreated MO‐LAS (100.0% ±4.4% vs. 47.8% ± 2.7%, *p* < 0.0001) (Figure 5E). Regarding cell migration, Sm4‐treated MO‐LAS cells exhibited an increased wound area after 24 h compared to the untreated cells (difference between the means of US vs. 156 μM Sm4 16.69% ± 2.699%, *p* < 0.0001, difference between the means of DMSO control vs. 156 μM Sm4 9.31% ± 2.699%, *p* < 0.0057). Additionally, a significant difference was observed between the DMSO treatment and the unstimulated control (difference between the means of US vs. DMSO control for 78 μM 7.38% ± 2.699%, *p* < 0.03) (Figure 5F,G). As a result, Sm4 treatment seems to impair cell functionality in MO‐LAS.

## 4 Discussion

In this study, we investigated the expression patterns and functional effects of SOX18 inhibition using Sm4 in HDLEC and lymphangiosarcoma tumor cell line (MO‐LAS). We detected high *Sox18* expression levels in HDLEC and MO‐LAS. HDLEC showed the highest *Sox18* expression among the tested cell lines, while MO‐LAS showed comparable mRNA levels to HDLEC. However, the SOX18 protein level in HDLEC was not significantly elevated compared to the other cell lines. Treatment with Sm4 resulted in a significant reduction of *Sox18* expression at both the mRNA and protein levels in HDLEC and MO‐LAS. Moreover, Sm4 treatment suppressed the expression of lymphatic phenotype key markers (*Prox1, Flt4*, and *Lyve1*) and reduced the migration and proliferation of both HDLEC and MO‐LAS cells.

The high expression of *Sox18* in HDLEC and MO‐LAS is consistent with previous studies in different tissue samples and cell lines that have implicated SOX18 as a key regulator of lymphatic endothelial cell development [15, 33] and in solid tumors [14, 33, 34]. The finding that HDLEC exhibited the highest *Sox18* expression compared to HDBEC and HUVEC further supports the notion that SOX18 is specifically enriched in lymphatic endothelial cells and is part of a specific lymphatic molecular profile.

The use of Sm4 as a SOX18 inhibitor has been explored in previous studies [29, 30]. Fontaine et al. demonstrated that Sm4 effectively inhibits SOX18 with the IC50 value reported with 5.2 μM [29] in fibroblasts. Our study extends these findings by showing that Sm4 treatment leads to a concentration‐dependent reduction in *Sox18* expression in HDLEC and MO‐LAS. Notably, to achieve the inhibitory effect on *Sox18* expression, higher concentrations of Sm4 were required in HDLEC (fifteenfold, 78 μM) and MO‐LAS (thirtyfold, 156 μM). These concentrations exceed those reported by Fontaine et al., with the distinction that Fontaine et al. used fibroblasts as a different cell line in their research.

It is essential to recognize that the effective Sm4 concentration may vary based on cell type, experimental conditions, and specific molecular pathways involved. While we confirmed that Sm4 concentrations applied to HDLEC and MO‐LAS did not affect cell viability, as indicated by MTT assays, the potential toxicity to other cell types within a tissue context remains uncertain. Our study was confined to in vitro experiments, highlighting the need for further investigations to assess the impact on various cell types within a tissue environment. Considering that MO‐LAS cells required a significantly higher concentration of 156 μM compared to 78 μM for HDLEC, the 156 μM concentration already compromised cell viability in HDLEC. The varying concentrations of Sm4 in HDLEC and MO‐LAS cells suggest differences in the cellular environment, receptor expression, or intracellular signaling pathways between healthy and cancerous lymphatic endothelial cells. Further investigation is necessary to elucidate the reasons for this differential sensitivity, including conducting dose–response studies and exploring potential compensatory mechanisms within the cells.

Furthermore, it is important to note that we have not determined the IC50 values for either cell line in this study. This was an initial test to evaluate whether Sm4 has any effect in general. Determining the IC50 should be the next step in our research to precisely quantify the inhibitory concentration of Sm4 for HDLEC and MO‐LAS cells.

The varying levels of SOX18 detected in different cancer types underscore the significance of this transcription factor in tumor progression, metastasis, and the formation of blood and lymphatic vessels within tumors. This highlights SOX18's crucial role and potential as a promising target in contemporary anticancer therapies [19, 22, 23]. Our study, demonstrating a reduction in *Sox18* expression upon Sm4 treatment in both HDLEC and MO‐LAS, suggests that SOX18 serves as a potential therapeutic target in regulating lymphangiosarcoma and cancer‐induced lymphatic metastasis biology. Additionally, our findings reveal that SOX18 inhibition by Sm4 results in a decrease in lymphatic phenotype key markers (PROX1, VEGFR3, and LYVE1), indicating a potential role for SOX18 in promoting cancer‐related lymphangiogenesis as well as lymphangiosarcoma development and progression.

Furthermore, Sm4 treatment resulted in reduced migration and proliferation of both HDLEC and MO‐LAS cells. These findings are consistent with previous studies that have demonstrated the involvement of SOX18 in the regulation of endothelial cell migration [29, 35, 36]. The diminished migration observed in our study suggests that SOX18 inhibition by Sm4 could impede the growth of lymphangiosarcoma cells and cancer‐related lymphangiogenesis. However, further studies are needed to confirm these effects. Our study aimed to identify Sm4 as a potential target, and future research should focus on detailed mechanistic studies and dose–response analyses.

The observed results provide valuable insights into the role of SOX18 and its inhibition in lymphatic endothelial and lymphangiosarcoma cells.

However, there are some limitations that should be acknowledged.

Firstly, our study focused on in vitro cell‐based experiments only, which may not fully recapitulate the complex microenvironment and interactions that occur in vivo. In vivo studies using animal models or patient‐derived samples would provide a more comprehensive understanding of the therapeutic potential of SOX18 inhibition in lymphangiosarcoma treatment and its effects on tumor growth, metastasis, and overall survival.

Another limitation of the present study results from the use of only one lymphangiosarcoma cell line (MO‐LAS) for our experiments. Lymphangiosarcoma is a rare and heterogeneous malignancy, and the response to SOX18 inhibition may vary among different subtypes or individual patients.

Additionally, we did not investigate how Sm4 reduces Sox18 expression. Furthermore, the specific molecular mechanisms through which SOX18 inhibition affects lymphatic phenotype markers, migration, and proliferation were not fully explored in this study. Future research should focus on elucidating the molecular pathways responsible for Sm4‐mediated Sox18 downregulation and understanding the downstream signaling pathways and molecular interactions involved in these processes.

While our study concentrated on Sm4's effect on *Sox18* expression, the necessity of exploring additional targets to fully grasp its broader impact on cell function and phenotype is crucial moving forward. Future experiments, including comprehensive proteomic and transcriptomic analyses, will be indispensable in uncovering other molecular targets of Sm4 and understanding their roles in influencing cellular behavior.

Despite these limitations, our study provides a foundation for future research on *Sox18* as a potential therapeutic target for lymphangiosarcoma cells and a more detailed understanding of its specific tumor biology. Further studies addressing the limitations mentioned will help advance our understanding of the therapeutic potential of SOX18 inhibition in the management of lymphatic metastasis and lymphangiosarcoma.

## 5 Conclusion

Our study demonstrates that *Sox18* is highly expressed in HDLEC and MO‐LAS cells and that its inhibition by Sm4 leads to a significant reduction in *Sox18* expression, suppression of lymphatic phenotype key markers, and decreased cell proliferation and migration. These findings support the potential of SOX18 as a therapeutic target for lymphangiosarcoma cells and warrant further in vitro investigation into the mechanisms and dose–response analyses in this context.

## Author Contributions

All authors had unrestricted access to the study data and bear accountability for the data's integrity and the accuracy of the data analysis. Conceptualization: Katja K. Koll and Christoph Hirche. Methodology: Katja K. Koll, Patrick A. Will and Christoph Hirche. Investigation: Katja K. Koll and Martin M. Zimmermann. Formal Analysis: Katja K. Koll and Martin M. Zimmermann. Resources: Ulrich Kneser. Writing – Original Draft: Katja K. Koll and Martin M. Zimmermann. Writing – Review and Editing: Patrick A. Will, Ulrich Kneser and Christoph Hirche. Visualization: Katja K. Koll, Martin M. Zimmermann. Supervision: Christoph Hirche.

## Conflicts of Interest

The authors declare no conflicts of interest.

## Supporting information


**Table S1.** Used Primers for qPCR.

## Data Availability

The data that support the findings of this study are available from the corresponding author upon reasonable request.
