# Supplementary material for: The Transcription Factor SOX18 Inhibitor Small Molecule 4 Is a Potential Treatment of Cancer‐Induced Lymphatic Metastasis and Lymphangiosarcoma
Source: Cancer Rep (Hoboken). 2025 Jan 10;8(1):e70110. doi: 10.1002/cnr2.70110 (PMC11726641; doi:10.1002/cnr2.70110)
Supplement: Supplementary file 1 — Table S1. Used Primers for qPCR. [file CNR2-8-e70110-s001.docx]

**Supplementary Table 1**

Supplementary Table 1. Used Primers for qPCR.

| **Genes** | **Forward sequence 5’ to 3’** | **Reverse sequence 3’ to 5’** |
| --- | --- | --- |
| *Gapdh* | CTCACTGTTCTCTCCCTCCG | CTAACGGCTGCCCATTCATT |
| *Sox18* | CTTCATGGTGTGGGCAAAG | GCGGCCGGTACTTGTAGTT |
| *Lyve1* | CTACTCCTCCTGCTCCAGCTT | ACCCAGCAGCTTCATTCTTG |
| *Prox1* | TGTCATCTCACCACCTGAGC | GATTGGGTGACAATCCTTCC |
| *Flt4* | AAGCTGGTGATCCAGAATGC | ACTTGTAGCTGTCGGCTTGG |

**Supplementary Table 1** – Abbreviation: *Glyceraldehyde-3-Phosphate Dehydrogenase* (*Gapdh*), *SRY-Box Transcription Factor 18* (*Sox18*), *lymphatic vessel endothelial hyaluronan receptor 1* (*Lyve1*), *Prospero Homeobox 1* (*Prox1*), *Fms Related Receptor Tyrosine Kinase 4* (*Flt4*).
